# Satellite Cells Senescence in Limb Muscle of Severe Patients with COPD

**DOI:** 10.1371/journal.pone.0039124

**Published:** 2012-06-13

**Authors:** Marie-Eve Thériault, Marie-Ève Paré, François Maltais, Richard Debigaré

**Affiliations:** Centre de recherche de l'Institut universitaire de cardiologie et de pneumologie de Québec, Québec, Canada; Clinica Universidad de Navarra, Spain

## Abstract

**Rationale:**

The maintenance of peripheral muscle mass may be compromised in chronic obstructive pulmonary disease (COPD) due to premature cellular senescence and exhaustion of the regenerative potential of the muscles.

**Methods:**

*Vastus lateralis* biopsies were obtained from patients with COPD (n = 16) and healthy subjects (n = 7). Satellite cell number and the proportion of central nuclei, as a marker of muscle regenerative events, were assessed on cryosections. Telomere lengths, used as a marker of cellular senescence, were determined using Southern blot analyses.

**Results:**

Central nuclei proportion was significantly higher in patients with COPD with a preserved muscle mass compared to controls and patients with COPD with muscle atrophy (p<0.001). In COPD, maximal telomere length was significantly decreased compared to controls (p<0.05). Similarly, minimal telomere length was significantly reduced in GOLD III–IV patients with muscle atrophy compared to controls (p<0.005). Minimal, mean and maximum telomere lengths correlated with mid-thigh muscle cross-sectional area (MTCSA) (R = 0.523, p = 0.005; R = 0.435, p = 0.019 and R = 0.491, p = 0.009, respectively).

**Conclusions:**

Evidence of increased regenerative events was seen in GOLD III–IV patients with preserved muscle mass. Shortening of telomeres in GOLD III–IV patients with muscle atrophy is consistent with an increased number of senescent satellite cells and an exhausted muscle regenerative capacity, compromising the maintenance of muscle mass in these individuals.

## Introduction

Skeletal muscle atrophy is observed in a variety of acute and chronic conditions [1]–[5] including chronic obstructive pulmonary disease (COPD) [6]. In COPD, the unintentional loss of muscle mass has a significant impact on quality of life [7] and is associated with premature death [8]. Many biochemical factors have been proposed to initiate and promote the development of skeletal muscle atrophy in COPD [9]. Satellite cell senescence impairing muscle regeneration capacity could be involved in this process [10]. To date, no studies have investigated the prevalence of satellite cell senescence in the context of muscle mass maintenance in COPD.

Satellite cells represent the primary source of myogenic precursor cells contributing to the processes of muscle mass maintenance, hypertrophy and repair after birth [11]. Under their quiescent state, satellite cells are found between the plasmalemma and the basal lamina on the surface of muscle fibre [12]. Upon activation, they proliferate into myoblasts and migrate to the requested site (reviewed by Charge [11]). Newly formed myoblasts will exit the cell cycle, differentiate into myotubes and fuse to an injured myofibre [13]. Newly formed myofibres exhibit a central nucleus and myogenic regulatory factors will subsequently help to migrate the nuclei toward the periphery to form a mature fibre [11]. As such, the proportion of central nuclei has been used as a marker of regenerative events within muscles [14], [15]. Senescent satellite cells are unable to enter this process and thus cannot contribute to muscle tissue maintenance [16].

Progressive shortening of satellite cell telomeres plays a significant role in their progression toward senescence thus contributing to premature proliferative arrest and poor tissue renewal. Telomeres are DNA–protein complexes (TTAGGG repeats) located at the ends of every eukaryotic chromosomes, promoting chromosomal and gene expression stability [17]. In the absence of telomerase [18], telomeric length decreases after every division cycle [19] because of the limitation of DNA polymerases in completing replication of linear DNA strands. Once telomere length reaches ∼5 kbp, cells enter an irreversible state of arrested growth and altered function, termed replicative senescence [19]. Even with slight telomerase activity in satellite cells [20], premature telomere shortening has been reported in atrophying models in which excessive number of regenerative events is present [21].

Considering that increased number of senescent satellite cells can directly limit the regenerative potential of muscle tissue, we speculated that the likelihood of finding senescent satellite cell in *vastus lateralis* would be significantly higher in patients with COPD and low muscle mass. To test this hypothesis, satellite cell and central nuclei numbers as well as telomere length in limb muscle specimens were quantified and compared between patients with COPD with or without muscle atrophy and healthy subjects.

## Materials and Methods

### Ethics statement

The research protocol was approved by the institutional ethics committee (Research ethics committee of the *Institut universitaire de cardiologie et de pneumologie de Québec*) and a signed informed consent was obtained from each subject.

### Subjects

Sixteen patients with Global Initiative for Chronic Obstructive Lung Disease (GOLD) stage III and IV COPD and seven healthy subjects with normal lung function, all males, were consecutively recruited for this study. Based on a power of 80% and type I error rate of 0.05 to find a significant difference of 3 central nuclei between atrophied and non-atrophied patients, 7 patients per group are needed. COPD diagnosis was based on a past smoking history (>10 pack-year) and pulmonary function test showing persistent airflow obstruction (forced expiratory volume in 1 s [FEV_1_] <50% of predicted value and FEV_1_/forced vital capacity [FVC] <70%) [22]. All patients with COPD were in a stable condition at the time of the study and were neither suffering from any other diseases nor using oral corticosteroids or any other medication that could alter satellite cells behaviour. Because low muscle mass is related to poor quality of life [23], reduced functional capacity [7] or survival [8], patients with COPD were subdivided based on the presence of muscle atrophy. In our study, muscle atrophy was defined as mid-thigh cross-sectional area (MTCSA) lower than 70 cm^2^. MTCSA <70 cm^2^ has been previously demonstrated to be associated with decreased survival [8].

### Pulmonary function, anthropometric measurements and body composition

Standard pulmonary function tests including spirometry, lung volumes, and carbon dioxide diffusion capacity were obtained in all subjects during the initial evaluation according to previously described guidelines [22]. Results were related to previously published normal values [24]. Height and weight were measured according to standardized methods [25]. Mid-thigh muscle cross-sectional (MTCSA) area was determined using computed tomography, as previously described [8].

### Muscle biopsy

One needle biopsy of the *vastus lateralis*, performed as described by Bergström and routinely done in our laboratory [26] was obtained in each participant. Muscle specimens were frozen in liquid nitrogen and stored at −80°C for future analysis.

### Immunofluorescence

Muscle section (10 µm) were fixed with ice-cold acetone and methanol 60/40 (v/v) at −20°C for 20 minutes, washed with PBS, incubated with a blocking solution (horse serum 1%) for 1 hour and then incubated with the primary antibody overnight at 4°C in a humidified chamber. The primary antibody Pax-7, (R&D Systems, Minneapolis, NE, USA) NCAM (R&D Systems) and laminin (DAKO, Glostrup, Denmark) excesses were cleaned with PBS, incubated with their specific secondary antibodies, a goat anti-mouse Alexa Fluor® 488 (R&D Systems) and a goat anti-rabbit Alexa Fluor® 546 (Invitrogen, Carlsbad, CA, USA) for 1 hour, washed with PBS and then incubated with DAPI for 15 minutes. Slides were analyzed and images were captured using a Nikon Eclipse E600 microscope (Nikon Corporation). Immunoflorescence were performed in duplicate on two distinct muscle cryosections. To ensure maximal image quality, pictures were taken within 24 hours after labelling. All the muscle samples where labelled during the same experimentation.

### Satellite cells and myonuclei labeling

To assess the number of satellite cells in muscle tissue, immunofluorescence was used. Satellite cells have heterogeneous expression of cellular markers resulting in a variety of subsets of cell populations. Thus, it is recommended to use more than one target to fully quantify satellite cells in a given sample [27]. Neural cell adhesion molecules (NCAM) and the transcription factor Pax7, two distinct proteins present in satellite cells, were used to assess the satellite cell number. Immunofluorescence was performed using a standard protocol against NCAM and Pax7 [27]. Laminin was used to delineate muscle fibres. Nuclei were labeled using DAPI. Nuclei located deep to the basal lamina and positive for NCAM or Pax7 were counted as satellite cells. The numbers of satellite cells was counted over 100 myofibres and reported as the number of satellite cells per 100 myofibres.

The presence of central nuclei was interpreted as a regenerative event since myonuclei incorporation is a specific step of muscle repair and satellite cells are responsible for 99% of muscle regeneration [28]. A central nucleus was defined as a committed cell being located inside the perimeter of the fibre delimited by laminin. Number of central nuclei per 100 myofibres is reported.

### Fibre typing and cross-sectional area

Muscle sections were stained using monoclonal anti-skeletal myosin fast (Sigma, Oakville, ON, Canada) at 4°C with 1∶200 dilution and VECTASTAIN Elite ABC system (Vector Laboratories, Burlington, ON, Canada). Peroxydase activity was detected and muscle fibres were classified according to the staining intensity: type I (non-stained) and type II (stained). All fibres were counted with an image analysing system (Image Pro Plus 4.5 for Windows, MediaCybernetics, Silver Spring, MD, USA) and classified to obtain the fibre-type composition for each subject. The mean muscle fibre cross-sectional area (CSA) was determined for each fibre type. The fibre CSA was calculated based on 75 randomly selected fibres for each sample [29].

### Genomic DNA extraction and Southern blot analysis

The intact genomic DNA extraction, digestion and migration were performed as previously described [30]. The intact genomic DNA obtained from muscle tissue was digested for 4 hours at 37°C in the presence of HinfI (0.2 units/µl) (New England Biolabs, Ipswich, Massachussetts) to generate DNA fragment containing telomere restriction fragments (TRFs). Three micrograms of digested genomic DNA and DNA ladder (1 kb and high molecular weight) were resolved by electrophoresis in 0,7% agarose gels that were denatured and neutralized before the transfer on nylon membrane. TRFs were detected by hybridization using a 32^P^-TTAGGG probe and visualized by autoradiography. The signal responses were analyzed by Image J.

### Telomere length analysis

The mean telomere length (L) was calculated by integrating the signal intensity above background over entire TRFs distribution as a function of TRFs length using the formula: L = Σ(ODi•Li)/Σ(ODi) where ODi and Li are the signal intensity and TRF length respectively at position L on the gel image. To determine the minimum and maximum value of telomere length in a homogenous way for all samples, the densitometric profile of TRF length was integrated over the distance of migration: minimum and maximum telomere length were corresponding to 5% and 95% of this integration, respectively. Minimum, mean and maximum telomere length values were determined for each sample from three independent gels as previously published [31].

Signal analysis generated three distinct measurements that were interpreted as follow: 1) minimal telomere length indicates the size of telomere structures found in dividing cells such as satellite cells. Thus, in satellite cells, shortening in minimal telomere length can be interpreted as their replicative history [32]; 2) mean telomere length represents the size of telomere structures in postmitotic myonuclei which have been incorporated into muscle fibres from birth to adulthood; 3) maximal telomere length is representative of the size of native telomere length as found in skeletal muscle cells [32]. Of these three measurements, a shorter minimal telomere length in a given sample can be interpreted as a high likelihood of finding a significant number of cells that have exhausted their regenerative potential in this tissue.

### Statistical analysis

Results are expressed as mean (± SEM). All data were normally distributed and statistical test performed accordingly. Differences for all variables between the three groups were analyzed using a one-way analysis of variance (ANOVA) except for telomere length analyses for which one-way analysis of covariance (ANCOVA) was performed to correct for age. Pearson linear regression analysis was performed to test relationship between variables. Differences were considered to be significant when p<0.05.

## Results

### Subjects characteristics

Anthropometric characteristics and pulmonary function data are provided in Table 1. Patients and healthy subjects did not significantly differ regarding age and body mass index. Ten patients had stage III, and the six remaining patients had stage IV disease according to the GOLD classification. As previously published, type I fibre distribution was significantly decreased in patients with COPD. Type II fibre distribution followed the reverse pattern as shown in Table 1. There was a significant decrease in type I fibre CSA in the group of patient with COPD and low muscle mass compared to controls and patients with COPD and preserved muscle mass as shown in Table 1.

**Table 1:** Subjects characteristics

	Controls	COPD	COPD
	(n = 7)	MTCSA >70 cm^2^	MTCSA <70 cm^2^
		(n = 9)	(n = 7)
**Age (years)**	67±2.7^a^	64±2.3^a^	70±1.7^a^
**BMI (kg/m^2^)**	27.2±1.7^a^	27.2±1.8^a^	22.3±1.7^b^
**FEV_1_, L**	3.1±0.2^a^	1.0±0.1^b^	1.0±0.2^b^ *
**FEV_1_, % predicted**	107.3±3.4^a^	34.0±2.5^b^	31.4±5.8^b^ *
**FVC, L**	3.9±0.3^a^	2.6±0.2^b^	2.8±0.2^b^ †
**FVC, % predicted**	105.6±4.5^a^	69.0±4.6^b^	71.7±8.6^b^
**FEV_1_/FVC, %**	79.3±1.5^a^	36.6±1.3^c^	33.1±3.9^c^ *
**DL_co_, (ml CO/min/mmHG)**	21.8±2.6^a^	14.7±1.1^b^	13.0±0.8^b^ †
**MTCSA (cm^2^)**	103.5±7.8^a^	85.5±3.3^b^	66.4±5.5^c^ *
*Type I fibre*			
**Distribution, %**	57.0±6.6^a^	32.8±3.6^b^	28.2±7.9^b^
**CSA, (µm^2^)**	5261±360^a^	4794±364^a^	4246±314^b^
*Type II fibre*			
**Distribution, %**	43.0±6.6^a^	67.2±3.6^b^	71.8±7.9^b^
**CSA, (µm^2^)**	4026±276	3731±203	3677±227

Definition of abbreviations: BMI = Body mass index; FEV_1_ = Forced expiratory volume in 1 second; FVC = Forced vital capacity; DLCO = Diffusing capacity of carbon monoxide; MTCSA = Mid-thigh cross-sectional area; CSA = Cross-sectional area. Values with different lower case letter are significantly different (p<0.05).

* p<0.001;

† p<0.01.

### Satellite cell quantification

**Figure 1:**
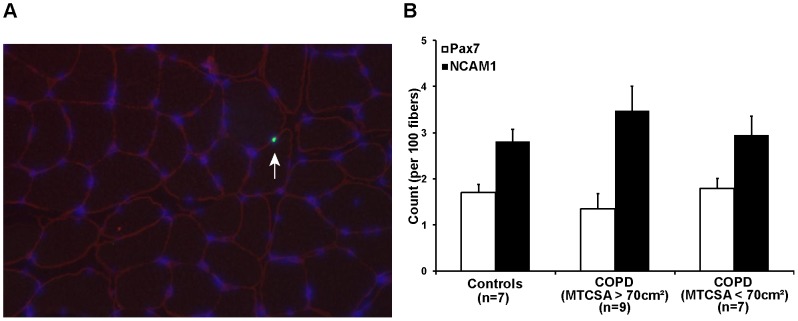
Satellite cells quantification. **A**) *Vastus lateralis* muscle cryosections were labeled for co-expression and localization of nuclear Paired box transcription factor 7 (green), DAPI (blue) and laminin (red). Cells positive for pax7 or NCAM (not shown) and DAPI located between the plasmalemma and the basal lamina were counted as satellite cells (white arrow). The number of satellite cells is expressed over 100 fibres and reported as a ratio. Ratio are calculated from 9 COPD and MTCSA >70 cm^2^, 7 COPD and MTCSA <70 cm^2^ and 7 healthy subjects. **B**) The numbers of nuclei having a positive label for Pax7 (white) and NCAM (black) were counted as satellite cells. Cells positive for pax7 or NCAM and DAPI located between the plasmalemma and the basal lamina were counted as satellite cells. The number of satellite cells is expressed over 100 fibres and reported as a ratio; ANOVA; p>0.05.

A representative *vastus lateralis* cryosections labeled for Pax7 and laminin is depicted in Figure 1A. The number of Pax7+ and NCAM+ satellite cells per 100 fibres did not differ between groups (Figure 1B).

### Central nucleus quantification

**Figure 2:**
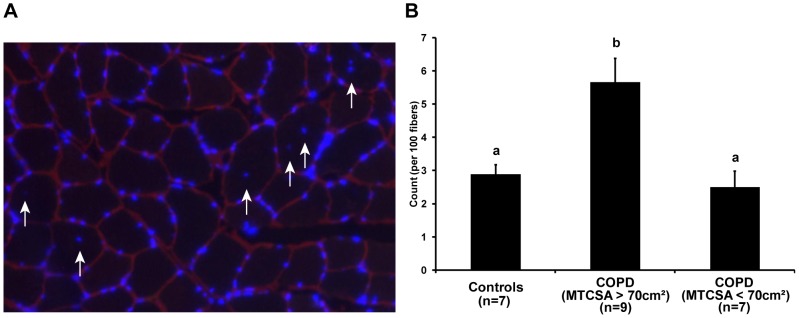
Central nucleus quantification. **A**) Cryosections were analyzed by labeling nuclei (blue) and laminin (red). Immuno-detection is shown for 10 µm skeletal muscle cryosections. The number of central nuclei (white arrows) is expressed over 100 fibres and reported as a ratio. Ratio are calculated from 9 COPD and MTCSA >70 cm^2^, 7 COPD and MTCSA <70 cm^2^ and 7 healthy subjects. **B**) The total numbers of central nuclei were quantified per 100 fibres for patients with COPD and MTCSA >70 cm^2^, patients with COPD and MTCSA <70 cm^2^ and for healthy subjects. Nucleus inside the perimeter of a given fibre delimited by laminin was counted as a central nucleus, expressed over 100 fibres and reported as a ratio. Distinct letter represents a statistically significant difference (ANOVA; p<0.05).

A representative cryosection labeled for nucleus and laminin is provided in Figure 2A. The number of central nuclei averaged per 100 muscle fibres was higher in patients with COPD with preserved muscle mass compared to patients with COPD and low muscle mass and to controls (5.64±2.95 vs 2.47±1.17 vs 2.86±1.58, respectively; p<0.05) (figure 2B).

### Telomere length

**Figure 3:**
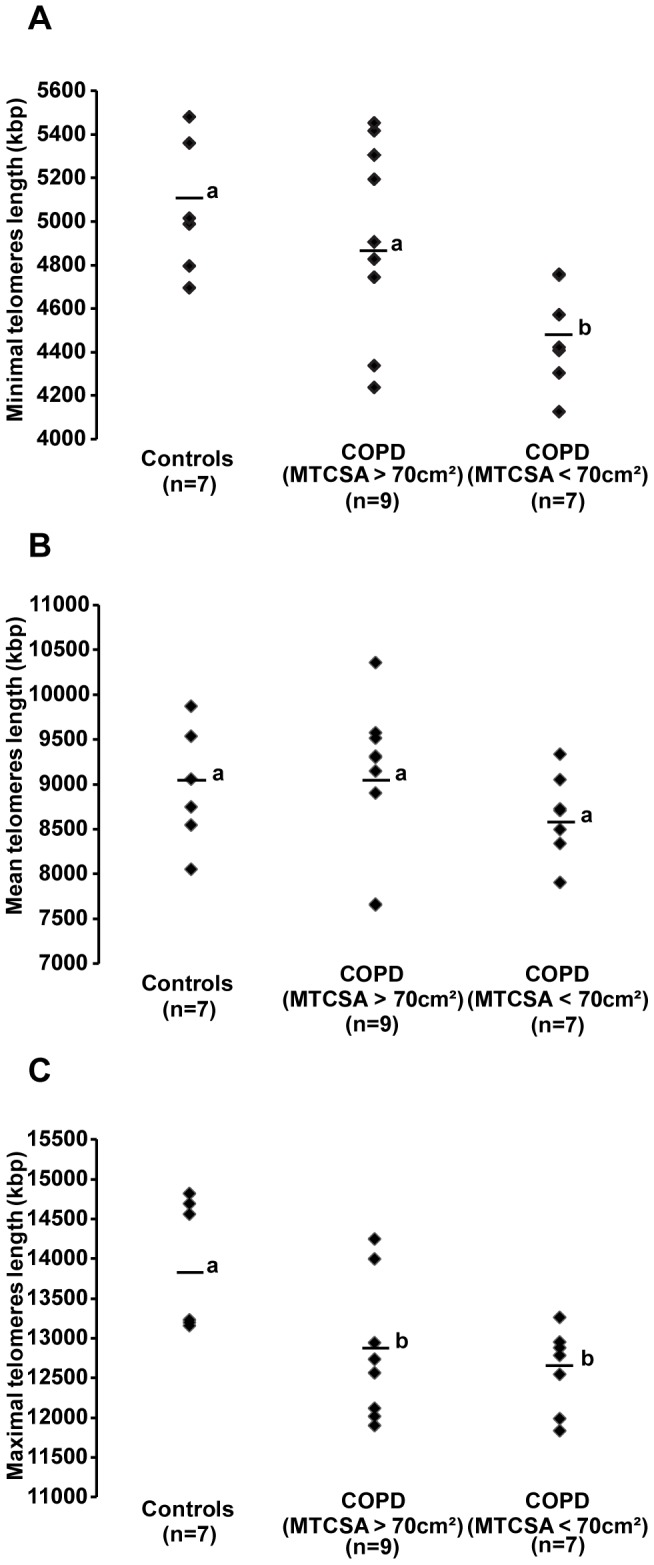
Telomere length. **A**) Minimal, **B**) Mean and **C**) Maximal telomere restriction fragment length of the *vastus lateralis* plotted in healthy subjects (n = 7), patients with COPD and MTCSA >70 cm^2^) (n = 9) and patients with COPD and MTCSA <70 cm^2^ (n = 7). The solid horizontal line represents the mean value for the group. Distinct letter represents a statistically significant difference (ANCOVA; p<0.05).

**Figure 4:**
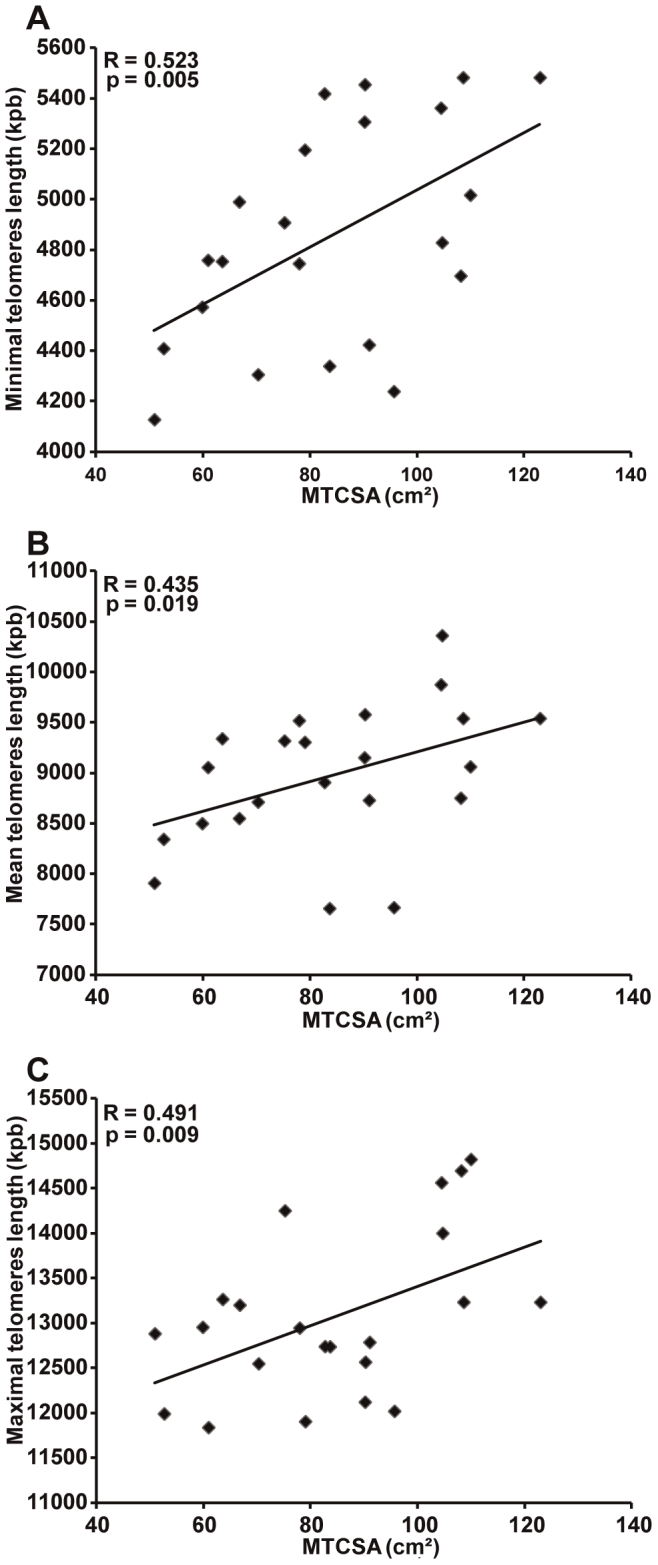
Minimal, mean and maximum telomere length correlation with MTCSA. **A**) Relationship between minimal telomere length and Mid-thigh Cross-Sectional Area (MTCSA) in patients with COPD and healthy controls. Telomere length significantly correlated with MTCSA in the studied population (r = 0.523; p = 0.005). **B**) Relationship between mean telomere length and Mid-thigh Cross-Sectional Area (MTCSA) in patients with COPD and healthy controls. Telomere length significantly correlated with MTCSA in the studied population (r = 0.435; p = 0.019). **C**) Relationship between maximum telomere length and Mid-thigh Cross-Sectional Area (MTCSA) in patients with COPD and healthy controls. Telomere length significantly correlated with MTCSA in the studied population (r = 0.491; p = 0.009).

Data for minimal, mean and maximal telomere lengths are plotted in Figure 3. Minimal telomere length was significantly shorter in patients with COPD and low muscle mass (4479±88 Kbp) compared to the other groups (COPD and a (MTCSA >70 cm^2^): 4938±149 Kbp and controls: 5119±123 Kbp; p<0.05). The mean telomere length was not significantly different between patients with COPD ((MTCSA >70 cm^2^): 9052±294 Kbp and (MTCSA <70 cm^2^): 8656±177 Kbp; p>0.05) and controls (9054±244 Kbp) even after analysis considering MTCSA. The maximal telomere length was significantly greater in controls (13846±302 Kbp) compared to patients with COPD ((MTCSA >70 cm^2^): 12811±277 Kbp and (MTCSA <70 cm^2^): 12611±197 Kbp; all p<0.05). Statistically significant correlations were found between minimal (figure 4A), mean (figure 4B) and maximal (figure 4C) telomere length with MTCSA (R = 0.523, p = 0.005, R = 0.435, p = 0.019 and R = 0.491, p = 0.009, respectively).

## Discussion

One of the most striking systemic consequences of COPD is the reduction in peripheral muscle mass resulting in muscle weakness, decreased functional capacity and reduced survival [33], [34]. Although several factors such as protein synthesis/degradation imbalance, hypoxia, inactivity, inflammation, and oxidative stress have been proposed to explain the initiation and the progression of muscle wasting in COPD [35], the potential contribution of impaired muscle regeneration has yet to be investigated. This was done in the present study by evaluating satellite cell number, occurrence of central nuclei and telomere length in peripheral muscle tissue of individuals with or without COPD. One of the major finding of this study is the significant decrease in minimal telomere length observed in patients with COPD and muscle atrophy compared to their COPD counterparts with better preserved muscle mass and healthy subjects. This observation is important because it suggests that the likelihood of finding senescent satellite cells is increased in patients with severe COPD and muscle atrophy. Supporting this assumption, presence of shorter telomeres in muscle tissue was correlated with lower MTCSA.

### Satellite cells in COPD

Regeneration of muscle tissue in a given individual relies on a sufficient number of competent satellite cells. As already shown, the abundance of satellite cells in peripheral muscle of patients with various degree of COPD had been demonstrated to be invariable [36]. In support of this study, this relative preservation in satellite cells number across these three groups as observed in our study suggests that COPD has an insignificant impact on the decline of the satellite cell population.

Despite the similar global satellite cells population in the 3 study groups, the number of central nuclei, a surrogate for the number of satellite cells engaged in a regenerative process, was significantly increased in patients with COPD and a preserved muscle mass compared to patients with COPD and muscle atrophy and to subjects with normal lung function. These results suggest that an accentuated number of regenerative events are taking place in the muscle tissue of a subset of patients with COPD having a preserved muscle mass. Similar results have been reported in diaphragm of patients with COPD [37]. However, tissue renewal was likely compromised in patients with COPD and muscle atrophy as indicated by a reduced number of central nuclei in these individuals compared to the other group of patient with COPD.

Discrepancies exist in the literature regarding the frequencies of central nuclei in muscle tissue of patient with COPD. Decramer et al. [14] has reported an increased number of central nuclei in steroid-induced myopathy in patients with moderate-to-severe COPD while Gosker et al. [15] did not find any difference between patients with a moderate-to-severe COPD and controls. This disparity could be attributed to the diversity of the population recruited and of the specific mechanisms involved in peripheral muscle dysfunction.

### Telomere length and muscle tissue

Similar to other tissues, replication of satellite cells throughout lifespan is marked by a progressive loss in telomere length defining a finite number of replicative events in which these cells can be engaged for muscle tissue regeneration [21]. For instance, increased number of replicative events following repetitive episodes of muscle injuries is paralleled by a faster telomere shortening in muscle altered by dystrophic conditions. In accordance, decreased telomere length was correlated with lower proliferative capacity in cultured satellite cells from patients with dystrophic muscles [38]. Telomere shortening in COPD has already been reported in alveolar type II epithelial cells [39] and blood leukocytes [40]–[42]. For the first time, the present report extends this finding to muscle tissue in which shorter telomere lengths was also found in patients with COPD when compared to healthy subjects.

The minimal telomere length shortening seen in patients with COPD and muscle atrophy is coherent with an increased satellite cells proliferation history in this population. In response to tissue damage, satellite cells increase their proliferation and are incorporated to myofibres through a complex and highly regulated myogenic program [11] a process during which the telomere length is shortened. The specific prevalence of limb muscle tissue injury throughout the progression of the COPD is unknown. However, presence of central nuclei was markedly increased in COPD with preserved MTCSA suggesting that, in this subpopulation of patients, the incidence of muscle damages resulting in activation of muscle repair is higher. Eventually, this would lead to the exhaustion of the replicative capacities of the satellite cells as indicated by the reduction in telomere length and to the inability to preserve muscle mass. The significant correlation between minimal telomere length and MTCSA supports this assumption.

Besides an accelerated satellite cell replication, several biochemical events may contribute to telomere shortening [43]–[45]. Because of its high guanine and cytosine content, a major target of reactive oxygen species, telomeric DNA is more susceptible to oxidative damage [46]. While most studies investigating the effects of oxidative stress on telomere shortening are *in vitro* [47], an *in vivo* link between oxidative stress and the rate of telomere shortening is emerging [48], [49]. During exercise, the production of reactive oxygen species, such as superoxide anions, hydrogen peroxide, and hydroxyl radicals is enhanced. Exercise is thus only be expected to increase the rate of oxidative damage to DNA and other cellular structures particularly when the capacity of the antioxidant defence mechanisms is exceeded in COPD [50]–[53]. In COPD, excessive oxidative stress has been reported in lower limb muscles at rest, after exercise and also during disease exacerbation [52]–[54]. Additionally, the increased level of antioxidant activity observed after exercise in healthy subjects is absent in patients with COPD [50], [55]. Thus, it is likely that accentuated oxidative stress, in addition to higher replicative rate history, contributes to the shortening of telomere length in muscle tissue of patients with COPD as reported in the present study.

### Methodological considerations

Despite a relatively modest sample size, a difference in telomere length was observed between groups. The positive correlation between telomere length and MTCSA is also reassuring about the validity of the results. Southern blot was used instead of other techniques such as PCR [41] to determine telomere length. This was done because Southern blot is reliable and has the advantage of providing three distinct length measurements that deliver complementary information about the replicative history of the cells. This methodology has also been extensively used in other studies investigating muscle tissue telomere length [21], [32]. Although telomere shortening was demonstrated, the mechanisms involved in this phenomenon remain unresolved. Determination of mechanisms and factors related to telomere shortening in patients with COPD was beyond the scope of this study. Because of the study design, analysis of the clinical relationship between functional status and telomere length was not possible. This issue should be addressed in subsequent studies.

### Conclusion

Even if the number of satellite cells was constant throughout the studied groups, the increased number of central nuclei observed is suggestive of an accentuated repair process within the leg muscle of patients with COPD and preserved muscle mass. An increase in damage/repair recurrence may exhaust the regenerative capacity of satellite cells leading to telomere shortening as exemplified by a shorter minimal telomere length in patients with COPD and muscle atrophy. Further studies on the intrinsic capacity of satellite cells to replicate and adopt a myogenic development are needed. Better understanding of the biological behaviour of satellite cells in COPD will be instrumental in designing pharmacological and exercise training interventions aimed at improving or maintaining muscle mass in this disease.
